# Human-machine collaboration in colorectal cancer screening: a narrative review of artificial intelligence-assisted colonoscopy

**DOI:** 10.3389/fmed.2026.1824724

**Published:** 2026-07-08

**Authors:** Chi Zhang, Jinguo Liu, Zhou Zhang, Liangliang Yu

**Affiliations:** Department of Endoscopy Center, Sir Run Run Shaw Hospital, Zhejiang University School of Medicine, Hangzhou, Zhejiang, China

**Keywords:** artificial intelligence, colonoscopy, colorectal cancer, early screening, human-machine collaboration, narrative review, quality control

## Abstract

Colorectal cancer (CRC) is a globally prevalent malignant tumor, and colonoscopy serves as the gold standard for its early screening and diagnosis. However, traditional colonoscopy highly depends on the experience of endoscopists, which is associated with limitations such as a relatively high miss rate and inadequate inspection quality control. This narrative review summarizes the application progress, clinical value, and existing challenges of artificial intelligence (AI) in colonoscopy. Currently, AI has formed three mature application directions, namely computer-aided detection, diagnosis, and quality control training, which extensively cover key links of colonoscopy, including bowel preparation assessment, cecal landmark identification, withdrawal quality monitoring, auxiliary lesion detection, and polyp character evaluation. Preliminary evidence suggests that AI can promote the standardization of colonoscopy procedures and the refinement of quality control indicators, potentially bridge the diagnostic gap between endoscopists with different levels of experience, and reduce the misdiagnosis of minor lesions. However, current studies predominantly report positive effects, and there is a lack of long-term outcome data, standardized methodology, and discussion of human-AI interaction dynamics. In conclusion, AI is expected to become an important auxiliary tool for the early screening and diagnosis of CRC, but further rigorous evaluation of its clinical value and collaborative workflow is needed.

## Introduction

Colorectal cancer (CRC) is a globally prevalent malignant tumor (1). According to data from the International Agency for Research on Cancer (IARC), there are over 1.926 million new CRC cases and approximately 930,000 deaths worldwide each year, ranking third in incidence and second in mortality among all malignant tumors (2). As a country with a high incidence of CRC, China reported over 517,000 new cases and more than 240,000 deaths in 2022, accounting for 27 and 25.7% of the global new cases and deaths, respectively, imposing a heavy medical and economic burden on families and society (3).

CRC is characterized by both an occult onset and preventability. The progression from precancerous lesions (e.g., adenomatous polyps) to advanced cancer typically takes a long period of 5–10 years, known as the “intervenable window period” (4). Early precancerous lesions, such as adenomatous polyps, can be completely cured through endoscopic minimally invasive resection, highlighting the indispensable role of colonoscopy in early screening (5).

However, the polyp miss rate remains a prominent issue in colonoscopy screening. The overall polyp miss rate in the general population ranges from 15 to 25%, with the miss rate of adenomatous polyps (precancerous lesions) being 10 to 20% (6). Missed polyps may lead to serious consequences, including CRC progression, increased treatment difficulty, and medical disputes (7). This problem is closely associated with polyp characteristics, examination quality, and equipment/technology (8). Therefore, optimizing patient education on bowel preparation, standardizing colonoscopy procedures, and reducing the polyp miss rate are important issues for gastroenterologists.

Although colonoscopy can significantly reduce CRC incidence by resecting all precancerous lesions, post-colonoscopy colorectal cancer (PCCRC) is still common, with an estimated incidence of 1 case per 1,000 patient-years (9). Most PCCRC cases are considered to be related to missed lesions during colonoscopy, and it is estimated that up to 25% of adenomatous lesions leading to CRC may be missed during examination. The causes of missed lesions are diverse, including failure to identify lesions that are fully visible on the endoscopic screen, inadequate bowel preparation, insufficient mucosal exposure, and poor technique during the withdrawal phase (10).

Although PCCRC incidence is the ultimate indicator of colonoscopy quality, its routine application is limited by practical operability. The adenoma detection rate (ADR) is the most commonly used marker of colonoscopy quality, defined as the proportion of individuals who undergo a complete colonoscopy with at least one adenoma detected and resected. Each 1% increase in ADR is associated with a 3% reduction in PCCRC risk. Other indicators, such as adenomas per colonoscopy (APC) and serrated polyp detection rate (SSLDR), also have great quality-control value (11).

In recent years, artificial intelligence (AI) has been deeply integrated into various aspects of colonoscopy. Currently, three mature application directions have been formed in digestive endoscopy, including computer-aided detection (CADe), computer-aided diagnosis (CADx), and quality control & training (12). A number of AI systems have entered clinical practice and obtained regulatory approval. The core value of AI lies in overcoming the limitations of human subjective judgment, enabling rapid processing of complex data, continuous performance optimization, and being free from the impact of fatigue and experience differences, thus providing a new solution for precision medicine (13).

Traditional endoscopy relies on physicians’ visual judgment and operational experience, which is associated with a high miss rate, poor diagnostic consistency, and insufficient operational standardization (14). The integration of AI technology has achieved breakthrough improvements in three dimensions: diagnostic accuracy, operational standardization, and clinical efficiency, and has formed multiple mature application systems in the screening and diagnosis of gastrointestinal diseases, moving from theoretical exploration to large-scale application (15).

This review is a narrative (non-systematic) summary of the literature based on the authors’ expertise and selective literature search. As such, it lacks a formal systematic methodology and may be subject to selection bias. The limitations of this approach are acknowledged in the Discussion.

## Methods

This narrative review synthesizes the current evidence on AI-assisted colonoscopy, focusing on clinical applications and human-machine collaboration. Given the rapid evolution and heterogeneity of the field, a narrative approach is appropriate to integrate diverse study types (randomized controlled trials, meta-analyses, prospective and retrospective cohort studies) into a clinically actionable overview. We searched PubMed from January 2015 to May 2026 using keyword combinations related to artificial intelligence, colonoscopy, colorectal cancer screening, CADe/CADx, bowel preparation, withdrawal time, and polyp detection/characterization. Reference lists of retrieved articles were hand-searched for additional relevant studies. Priority was given to high-quality studies reporting quantitative performance metrics relevant to clinical practice, such as adenoma detection rate, polyp miss rate, sensitivity, specificity, and area under the curve. Two authors independently screened titles and abstracts, and disagreements were resolved by consensus with a third author. Data were extracted thematically into five domains: bowel preparation assessment, cecal landmark detection, withdrawal quality monitoring, lesion detection, and polyp characterization. No formal quality scoring or meta-analysis was performed due to heterogeneity in outcome definitions and study designs; instead, we critically appraised each study’s design, sample size, validation strategy, and risk of overfitting. Limitations of the narrative approach, including potential selection bias and lack of quantitative synthesis, are acknowledged in the Discussion.

The initial database search yielded 847 records. After removing duplicates and screening titles/abstracts, 226 full-text articles were assessed. Ultimately, 77 studies met the inclusion criteria and were included in this narrative review, comprising 25 randomized controlled trials, 18 meta-analyses or systematic reviews, and 28 prospective/retrospective cohort or diagnostic accuracy studies. As a narrative review, we did not aim for exhaustive coverage of all published literature. Instead, we prioritized high-impact, recent, and methodologically diverse studies to provide a clinically actionable overview.

## Artificial intelligence-assisted colonoscopy

### Artificial intelligence improves the quality of bowel preparation

Adequate bowel preparation is essential for achieving optimal visualization of the colonic mucosa (16). However, inadequate bowel preparation is associated with an increased need for short-term repeat colonoscopy, prolonged procedural duration, elevated procedural risks, and unnecessary additional costs (17). Prior to colonoscopy, physicians often assess bowel preparation quality based on the cleanliness of the patient’s last bowel movement before the procedure. Patients typically provide a vague, approximate score based on subjective judgment, which frequently deviates substantially from the actual adequacy of bowel preparation (18).

Zhu et al. developed an AI-based prediction system for bowel preparation quality among outpatients prior to colonoscopy. This system can evaluate images of stool uploaded by patients and accurately predict the adequacy of bowel preparation, with an accuracy of 95.15% and a specificity of 97.25%. Subsequently, a smartphone application was developed based on this system to provide patients with personalized bowel preparation instructions according to the AI-predicted preparation status. In the next phase of the study, a prospective, multicenter, randomized trial of outpatients scheduled for colonoscopy revealed that the AI app group had a significantly higher rate of adequate bowel preparation (88.54% vs. 65.59%) and better adherence to pre-colonoscopy dietary restrictions and laxative administration than the control group (19).

During colonoscopy, most scoring systems for the assessment of bowel preparation rely on the subjective evaluation of endoscopists, with the Boston Bowel Preparation Scale (BBPS) being the most widely used worldwide (20); some institutions adopt the Aronchick Scale or the Ottawa Bowel Preparation Scale (21). All these scales are affected by the endoscopist’s experience, fatigue, and workload, and documentation based on memory may lead to inaccuracies (22). These limitations can be addressed by AI algorithms, which enable real-time, automated, and objective evaluation of bowel preparation quality (23).

Low et al. developed an AI model for the assessment of bowel cleanliness trained on 2,361 static colonoscopic images, achieving an accuracy of 94.0% on still images, with an overall agreement rate of 85.3% between endoscopists and the AI model in clinical applicability studies (24). Lee et al. developed an AI/convolutional neural network (CNN) algorithm based on colonoscopic videos, which achieved 100% sensitivity for detecting inadequate bowel preparation, with a high level of agreement with expert raters (76.7–83.3% for ten-second clips and 68.9–89.7% for complete videos) (25). Zhou et al. developed a novel bowel preparation assessment system named ENDOANGEL using a deep convolutional neural network (DCNN). In a human-machine competition involving 120 images, ENDOANGEL achieved an accuracy of 93.33%, outperforming all participating endoscopists; its accuracy was 80.00% for 100 bubble-containing images and 89.04% for 20 colonoscopic videos. The system continuously displays the cumulative percentage of images with different BBPS scores and prompts for scoring every 30 s during withdrawal, demonstrating reliable clinical applicability (26). The research team further optimized the DCNN model to develop an AI system for automatically calculating the proportion of bowel segments with BBPS 0–1 scores (e-BBPS score) during withdrawal, and found a significant negative correlation between the e-BBPS score and ADR (Spearman rank correlation coefficient [rs] = 0.976, *p* < 0.01). A follow-up study re-performing colonoscopies on patients with inadequate bowel preparation classified by the system showed a significantly higher detection rate of small adenomas (≤5 mm) in this cohort (35.71% vs. 13.19%, *p* = 0.0056), confirming the clinical value of the system (27).

Collectively, AI enables automatic scoring of bowel cleanliness through deep learning, replacing traditional subjective manual judgment, reducing inter-observer variability, standardizing BBPS scoring, improving the basic quality of colonoscopy, and indirectly enhancing ADR. Although AI-based scoring systems improve objectivity, their performance deteriorates in extremely poor bowel preparation (BBPS 0–1) or with excessive mucus, leading to false-negative or false-positive assessments. Such errors may result in repeated procedures or unnecessary interventions, a point further discussed in the Discussion section.

### Artificial intelligence for the detection of cecal landmarks

PCCRC refers to new CRC diagnoses within the recommended surveillance interval after a negative colonoscopy, mostly caused by missed lesions, incomplete resection, or rapid tumor progression, and serves as a key indicator for evaluating colonoscopy quality (28). The cecal intubation rate (CIR) is a key quality indicator of colonoscopic completeness and is directly linked to PCCRC risk; patients examined by endoscopists with a high CIR have a lower risk of PCCRC (29).

A complete colonoscopy is defined as successful intubation into the cecum with identification of the ileocecal valve and appendiceal orifice as anatomical landmarks (30). The American Society for Gastrointestinal Endoscopy-American College of Gastroenterology (ASGE-AC) and the European Society of Gastrointestinal Endoscopy (ESGE) released 2024 updated colonoscopy quality targets, recommending a CIR of ≥95% for routine screening colonoscopy and ≥90% (ideally ≥95%) for diagnostic/therapeutic repeat colonoscopy, with mandatory photographic documentation of the ileocecal valve, appendiceal orifice, and cecal folds (31, 32). The 2024 UK Bowel Cancer Screening Programme (BCSP) standards set a higher CIR threshold of ≥92% for diagnostic and therapeutic colonoscopy, reflecting the critical importance of CIR (33).

Approximately 60–70% of CRC and the vast majority of advanced adenomas are located in the right-sided colon (cecum, ascending colon, and proximal transverse colon) (34). Patients with failed cecal intubation exhibit a 2.5-fold higher risk of CRC and higher CRC-related mortality, mainly due to missed right-sided colonic neoplasia (35). Complete colonoscopy significantly reduces the overall incidence of CRC, with a more pronounced risk reduction for left-sided colon cancer and rectal cancer (36). However, CIR in actual clinical practice may not meet these benchmarks (37). Conventional AI systems for detecting cecal landmarks are easily affected by bowel preparation quality (38). To address this limitation, Low et al. extracted 13,222 colonoscopic images at the inspection endpoint (6,663 with appendiceal orifice [AO], 6,663 without AO), graded by BBPS scores 1–3, and trained a DCNN model. The algorithm classified images with or without AO with an accuracy of 94% and an AUC of 0.98, achieving AO detection rates of 97.5, 95.3, and 95.7% in images with BBPS scores of 1, 2, and 3, respectively, and 95.4 and 97.5% in adequately (BBPS ≥2) and inadequately (BBPS <2) prepared bowel images, respectively (39).

Taghiakbari and colleagues employed a deep learning model to analyze 25,444 frames from complete colonoscopy videos of 318 patients, with the ileocecal valve, AO, polyps, and normal mucosa as classification targets. The AI achieved a frame-level accuracy of 86.4% for both the ileocecal valve and AO, and an 85.7% patient-level rate for simultaneous detection of both landmarks, confirming reliable identification of cecal anatomical landmarks for automated quality control (40). A research team from Zhongshan Hospital, Fudan University, constructed a deep-learning-based model for ileocecal valve detection trained and validated on 18,962 colonoscopic images, achieving an identification accuracy of 95.27% (AUC not reported) and 97.1% in adequately prepared bowel images (41).

### Artificial intelligence-assisted high-quality colonoscopic withdrawal

High-quality colonoscopic withdrawal and adequate withdrawal duration (WT) are the cornerstone of high-quality colonoscopy, correlated with a reduced tumor miss rate (42). WT is a key factor influencing ADR; a prolonged WT correlates with a higher ADR and reduced PCCRC risk (43). All international guidelines recommend a minimum WT of 6 min (target of 8 min or more), with prolonged WT for ADR below 30% (44).

However, WT frequently fails to reach the recommended duration in clinical practice: a Chinese multicenter study revealed that 80% of colonoscopic procedures had a WT of less than 6 min, potentially due to inadequate staffing, heavy workload, and lack of quality control (45). WT also has limitations, as it does not consider withdrawal technique; suboptimal practices (inconsistent speed, unstable luminal view) compromise its reliability as a quality metric, with high-performing endoscopists achieving high ADR in a short duration and low-performing endoscopists requiring 11–14 min (46, 47).

Multiple AI-based systems have been developed to optimize WT via real-time feedback, improving withdrawal standardization. Gong and colleagues developed ENDOANGEL (DCNN + perceptual hashing algorithm) for real-time automatic WT monitoring, which alerts for excessively fast withdrawal and displays pre-slip images until reinsertion to the matching position. A randomized controlled trial (RCT) showed that the ENDOANGEL group had a significantly higher ADR in both intention-to-treat (ITT) (OR, 2.30; 95% CI, 1.40–3.77) and per-protocol (PP) (OR, 2.18; 95% CI, 1.31–3.62) analyses, with no significant difference in WT between groups (48).

Lux and colleagues established a multi-class DCNN model to address the deficiencies of traditional manual recording (mean WT measurement error of 4.2 min, key image retention rate of merely 68.5%). Trained on 1,300 videos and 10,557 key frames from 9 centers and validated on 100 videos from 5 centers, the AI achieved a mean absolute error of only 2.2 min for WT (correlation coefficient = 0.89, error < 3 min in 82% of cases, significantly better than manual recording [45%]). The model had recognition accuracies of 94.0% for the ileocecal valve and 92.3% for the AO, increasing the complete documentation rate of key images to 98.0% and report completeness to 99.0% without increasing endoscopist workload, and reducing examination time by 3.5 min per case (49).

Lui and colleagues employed a deep-learning-based model to calculate effective withdrawal time (EWT), defined as WT excluding frames with inadequate/invalid views, trained and optimized on 17,118 colonoscopic images. A retrospective analysis of 944 examinations and prospective validation of 89 cases showed the AI system achieved an EWT measurement accuracy of 92.1%, with high consistency with manual assessment (ICC = 0.969, r = 0.972) and a mean absolute error of only 0.18 min, with no latency and adaptability to various bowel preparation conditions. The ADR (37.5%) and polyp detection rate (PDR) in the EWT ≥ 6 min group were significantly higher than those in the EWT < 6 min group (19.0, 45.2%), with each 1-min increase in EWT associated with a 49% improvement in ADR (adjusted OR, 1.49; 95% CI, 1.36–1.65). This verifies EWT as a more precise quality control marker for standardized withdrawal management (50).

Colonic mucosal folds form visual blind spots during withdrawal, and no quantitative criteria exist for fold examination quality (FEQ) in clinical practice (51). Liu and colleagues established a DCNN-based AI-assisted system to address the subjectivity and non-uniformity of traditional FEQ evaluation, validated on 103 withdrawal videos from 11 endoscopists with manual ratings from 3 senior endoscopists as the gold standard. The AI-FEQ score was highly correlated with expert ratings (r = 0.871, *p* < 0.001) and significantly positively associated with the physician’s historical ADR and WT (r = 0.852, 0.727; all *p* < 0.05). AI assistance significantly enhanced the FEQ of endoscopists with low ADR (ADR < 25%), increasing their AI-FEQ score from 0.23 to 0.29 and expert rating from 11.67 to 14.00 (all *p* < 0.001), providing a novel approach for standardized, real-time withdrawal quality control (52).

### Artificial intelligence-assisted colonoscopy for improved lesion detection

AI incorporation into colonoscopy can enhance colorectal lesion detection by overcoming human perceptual bias, inattention, and fatigue—key factors for diagnostic errors and missed findings (53). CADe systems based on deep learning identify suspicious lesions in real time, serving as a permanent second observer for endoscopists, marking lesions for further evaluation, improving colonoscopy quality, and enhancing the detection/resection of premalignant lesions for CRC prevention (54).

A random-effects meta-analysis of 28 RCTs involving 23,861 patients demonstrated that AI-assisted colonoscopy was associated with a 20% increase in ADR and a 55% reduction in adenoma miss rate (AMR), mainly attributable to an increased detection rate of diminutive lesions; no significant differences were observed in the detection/miss rate of sessile serrated lesions. AI-assisted colonoscopy prolonged mean WT by 9 s and increased the hyperplastic polyp resection rate by 39%, potentially related to AI’s limited ability to differentiate hyperplastic polyps from adenomatous polyps (55). Another meta-analysis of seven RCTs investigating AI’s effect on AMR and polyp miss rate (PMR) found that the AI group had significantly reduced AMR and PMR, with reduced miss rates of sessile serrated lesions and diminutive adenomas (no significant effect on advanced adenomas). The average number of polyps and adenomas detected during re-examination was lower in the AI group, with no prolonged WT (56).

A prospective, quasi-randomized, controlled, multicenter trial in Denmark enrolled 795 participants assigned to AI-assisted (GI Genius, Medtronic) or conventional colonoscopy groups, with endoscopists categorized as experts (≥1,000 colonoscopies) or non-experts (<1,000 colonoscopies). The AI group had a significantly higher ADR (59.1% vs. 46.6%, *p* < 0.001), with a significant increase among experts (59.9% vs. 47.3%, *p* < 0.002) but not non-experts; AI significantly improved ADR in screening colonoscopy (74.4% vs. 58.1%, *p* = 0.003). The AI group also had a higher PDR (69.8% vs. 56.2%, *p* < 0.001), with no significant difference in the non-neoplastic resection rate (NNRR) (57).

A prospective RCT of automatic polyp detection using a SegNet-based DCNN enrolled 1,058 subjects in AI-assisted and conventional groups, showing the AI system significantly increased ADR (29.1% vs. 20.3%, *p* < 0.001) and the average number of adenomas per patient (0.53 vs. 0.31, *p* < 0.001). The AI group detected more adenomas < 5 mm (185 vs. 102; *p* < 0.001), with no statistical difference in adenomas > 5 mm (77 vs. 58, *p* = 0.075), and a significantly higher number of hyperplastic polyps (114 vs. 52, *p* < 0.001) (58).

Recent advances have addressed a core limitation of conventional CADe systems for colonic polyps, which require on-premises deployment of high-performance hardware (e.g., FPGAs/GPUs) that hinders system updates and locks healthcare institutions into legacy infrastructure, with cloud-based CADe solutions emerging to overcome these constraints; the multicenter randomized controlled EAGLE (Evaluation of AI for detection of Gastrointestinal Lesions in Endoscopy) trial, designed to assess the efficacy of Olympus CADDIE, a cloud-deployed real-time AI-assisted CADe system, for colorectal neoplasia detection during colonoscopy, enrolled 841 patients randomized 1:1 to the AI-assisted CADe group or standard-of-care (SoC) colonoscopy group (performed by 22 endoscopists across 8 centers in 4 European countries), and demonstrated that the CADe group achieved a 33% relative increase in mean adenomas per colonoscopy (APC, 0.82 vs. 0.62, *p* = 0.01), a 7.3% absolute increase in adenoma detection rate (ADR, 43.2% vs. 35.9%, *p* = 0.03), with no significant between-group difference in per-polyp adenoma rate (PPA, 53.9% vs. 53.4%) indicating no excess unnecessary resections, alongside 93, 57 and 230% relative increases in the detection rates of large adenomas, non-polypoid adenomas and sessile serrated lesions (SSLs), respectively (all *p* < 0.01) (59).

AI-assisted colonoscopy is well-validated to improve ADR, yet critical gaps remain in characterizing real-time endoscopist-AI collaborative dynamics, with prior eye-tracking studies largely limited to retrospective video reviews. Zhu et al. established the first open-source eye-tracking dataset for colonoscopic endoscopist-AI teaming, including two complementary subsets: a retrospective dataset from 26 participants across 4 proficiency tiers reviewing 60 colonoscopy clips (41 with polyps), and a prospective real-time dataset of 80 full colonoscopies (43 with CADe assistance, 37 standard-of-care). Validation confirmed the dataset’s robustness, and revealed CADe universally shortened polyp detection reaction time with a pronounced benefit for less experienced operators, providing a foundational open resource for AI-assisted endoscopy research (60).

In summary, AI-assisted systems conduct real-time, continuous, and fatigue-free image scanning of the intestinal mucosa through deep learning, accurately identifying diminutive adenomas, flat lesions, and sessile serrated lesions that are easily overlooked in traditional endoscopy. However, in addition to the reported benefits, CADe systems generate false-positive alerts. These alerts may disrupt the clinical workflow and lead to alarm fatigue, potentially desensitizing endoscopists to genuine alerts.

A controlled lab study by Van Berkel et al. involving 21 endoscopists demonstrated that false-positive AI recommendations significantly prolong decision-making time, and that the type of medical content displayed affects how quickly endoscopists respond to AI suggestions (61). Beyond controlled settings, a large-scale systematic review and meta-analysis (13 studies, 1,538 gastroenterologists and trainees) synthesized real-world provider attitudes and identified three major barriers to the clinical adoption of AI-assisted colonoscopy (62). First, excessive false-positive alerts were consistently cited as a primary concern, as they increase cognitive load, interrupt procedural flow, and may lead to unnecessary polypectomies and pathology examinations without corresponding patient benefit. Second, deployment and operational costs—including hardware acquisition, software licensing, system maintenance, staff training, and subsequent upgrades—were perceived as a substantial economic barrier by approximately half of the surveyed physicians (52, 95% CI 24–79%). Third, the absence of authoritative clinical guidelines from major professional societies was noted as a critical impediment, leaving endoscopists without standardized protocols for AI integration and quality assurance. Additionally, 38% of respondents expressed concern about liability for misdiagnoses potentially attributable to AI recommendations (95% CI 9–73%), highlighting unresolved medico-legal issues. Quantifying these adverse effects and developing strategies to mitigate false-positive burdens, reduce implementation costs, and establish clear regulatory and ethical frameworks remain important priorities for translating the technical promise of AI into sustainable, high-quality clinical practice.

### Artificial intelligence-assisted evaluation of polyp features

The diagnosis of polyp types largely relies on magnifying colonoscopy (up to ×520 magnification), which clearly visualizes lesion cellular atypia and glandular architecture. Combined with narrow-band imaging (NBI), this technique enables non-staining visualization of lesion surface microvasculature, achieving “optical biopsy” and avoiding contrast staining risks, with diagnostic performance superior to conventional magnifying NBI (×80–100 magnification) (63).

However, magnifying colonoscopy is highly dependent on endoscopist experience; interpreting EC-NBI microvascular and microglandular structures is challenging, especially for differentiating adenomas and invasive carcinoma (64). Junior physicians cannot achieve the same diagnostic accuracy as experienced specialists, resulting in poor inter-observer agreement and relatively high rates of missed lesions and misdiagnosis (65). Existing CADx models are limited to benign/malignant binary classification and cannot distinguish non-neoplastic lesions, adenomas, and invasive carcinomas, failing to meet clinical requirements for refined diagnosis and treatment decision-making (66).

Wang et al. developed a CAD model for multi-class classification of colorectal lesions (non-neoplastic lesions, adenomas, invasive carcinoma) using EC-NBI images, with 32,142 images of 406 lesions in the training set, 498 lesions in the internal validation set, and 2,749 images of 152 lesions in the external validation set. In image-level internal validation, the AUC values were 0.939 for non-neoplastic lesions, 0.917 for adenomas, and 0.969 for invasive carcinoma; in external validation, the AUC values were 0.837, 0.827, and 0.908, respectively. Lesion-level performance was superior, with silhouette coefficients of 0.8116 and 0.6673 in internal and external validation, respectively. The silhouette coefficient is a measure of how similar a lesion is to its own class compared to other classes—higher values indicate better internal consistency. AI improved the diagnostic performance of physicians with different experience levels: junior physicians’ diagnostic accuracy increased by 18.6–22.3% (Kappa value elevated from 0.58 to 0.82), moderate-experience physicians by 10.2–13.5%, and senior physicians by 5.1–7.8% (67).

In a study of 118 colorectal lesions, Kominami et al. compared the performance of magnifying NBI with that of CADx. The results demonstrated a 97.5% concordance rate between endoscopic diagnosis and CADx output. The accuracy, sensitivity, specificity, positive predictive value (PPV), and negative predictive value (NPV) of this CADx system were 94.9, 95.9, 93.3, 95.9, and 93.3%, respectively (68).

In contrast, Song et al. employed a deep-learning model for real-time classification of polyps in NBI images. Their CADx system was capable of categorizing polyps into three groups: serrated polyps, benign adenomas, and mucosal/superficial submucosal carcinoma versus deep submucosal carcinoma. The corresponding area under the receiver operating characteristic curve (AUROC) values were 0.93–0.95, 0.86–0.89, and 0.89–0.91, respectively. The overall diagnostic accuracy ranged from 81.3 to 82.4%, which was superior to that of novice endoscopists and comparable to that of expert endoscopists. When CADx was used to assist novice endoscopists, the concordance between actual and predicted polyp histology was significantly improved (kappa value increased from 0.368 to 0.655), and diagnostic accuracy rose from 63.8–71.8% to 82.7–84.2% (69).

A meta-analysis including 18 studies (3 prospective, 15 retrospective, totaling 7,680 polyp images) evaluated the performance of CADx models in predicting polyp histology. The pooled sensitivity was 92.3% (95% confidence interval [CI] 88.8–94.9%), specificity was 89.8% (95% CI 85.3–93.0%), and AUROC was 0.96 (95% CI 0.95–0.98). Six of these studies compared CADx with non-expert endoscopists and found that CADx achieved significantly higher accuracy in predicting polyp histology (AUC 0.97 vs. 0.90, respectively; *p* < 0.01) (70).

Furthermore, CADx has been investigated in combination with endoscopic biopsy techniques using a special contact microscopy colonoscope with 520 × optical zoom, combined with mucosal staining to visualize cellular structures. A single-center, open-label, prospective study demonstrated that, when endoscopic biopsy was combined with CADx, the NPV for rectal and sigmoid adenomas <5 mm was 93.7–96.4% under methylene blue staining and 95.2–96.5% under NBI, meeting the 90% threshold for “resect and discard” recommended by the American Society for Gastrointestinal Endoscopy (ASGE) PIVI criteria (71, 72).

Rodriguez-Diaz et al. adopted a CADx model that uses semantic segmentation and augmented visualization to generate a histology map over the polyp surface. Trained on 740 high-magnification NBI images (286 patients, 607 polyps, >65,000 subregions), the model distinguished neoplastic from non-neoplastic polyps with a sensitivity of 96%, specificity of 84%, and NPV of 91% (for polyps ≤5 mm: sensitivity 95%, specificity 84%, NPV 91%). This augmented visualization approach improves interpretability and transparency of AI-based optical biopsy, providing user-friendly guidance for clinical decision-making (73).

Collectively, the literature on CADx for colorectal polyps reveals several overarching trends and persistent gaps. First, regarding diagnostic performance, the pooled sensitivity (92.3%) and specificity (89.8%) from 18 studies indicate that deep learning models can achieve high accuracy for binary (neoplastic vs. non-neoplastic) classification, often matching or exceeding that of expert endoscopists (70). However, performance drops substantially in multi-class tasks (e.g., distinguishing adenomas from sessile serrated lesions or early invasive carcinoma), as seen in the lower AUC values for adenoma classification (0.86–0.89) in some models (69, 71). Second, regarding generalizability, a critical concern is that most models have been developed and validated on retrospective, high-quality, still images (typically captured under ideal conditions with magnification and NBI). Their performance in real-time, low-quality, or motion-blurred video streams—where bowel preparation is suboptimal or polyps are small and flat—remains largely unproven. Only a few studies have prospectively validated CADx systems in live colonoscopy, and external validation across different endoscopy systems (e.g., different NBI generations, white-light only) is rare. Third, the clinical impact of CADx has been demonstrated primarily through improvements in diagnostic accuracy of less experienced endoscopists, narrowing the gap between novices and experts (67, 69). This suggests that CADx may be most valuable as a training and decision-support tool in community settings where expert pathologists or endoscopists are not immediately available. Fourth, unresolved controversies include the optimal confidence threshold for “resect-and-discard” strategies, the management of low-confidence predictions, and the lack of prospective cost-effectiveness analyses. Furthermore, most CADx models remain “black boxes” with limited interpretability, hindering clinician trust and regulatory acceptance. Additionally, most CADx models have been evaluated on still images rather than continuous video streams; real-time constraints such as frame processing rate, latency, and motion artifacts have not been systematically addressed. Fifth, future research should prioritize (i) prospective, multicenter trials that test CADx in real-time colonoscopy across diverse patient populations and endoscopy systems; (ii) integration of CADx with CADe into unified AI platforms that both detect and characterize lesions; (iii) development of explainable AI methods that provide visual or textual justifications for predictions; and (iv) health economic evaluations to determine whether the additional accuracy translates into reduced unnecessary polypectomies, lower pathology costs, or improved patient outcomes.

## Discussion

Multiple randomized controlled trials and meta-analyses have consistently demonstrated that AI-assisted colonoscopy improves ADR and reduces AMR, particularly for diminutive polyps (55, 56). There is also broad consensus that AI-based bowel preparation scoring and cecal landmark detection significantly reduce inter-observer variability (26, 27, 39, 40). Despite these encouraging findings, no consensus has yet been reached regarding the optimal model of human–AI interaction, the long-term impact of AI on interval CRC incidence, or the cost-effectiveness of these systems. Moreover, although the literature predominantly reports positive effects, several important controversies remain. First, it is unclear whether the observed increase in detection of diminutive polyps translates into a clinically meaningful reduction in CRC mortality, as long-term follow-up studies are still lacking (7, 28). Second, there is a potential risk of skill degradation when endoscopists rely heavily on AI; emerging evidence now suggests that such degradation may indeed occur (see below). Third, the clinical implications of a 9-s prolongation of withdrawal time and a 39% increase in hyperplastic polyp resection should be distinguished: the additional 9 s is unlikely to have meaningful clinical consequences, whereas a 39% increase in hyperplastic polyp resection may lead to unnecessary polypectomies, additional pathology costs, and patient anxiety without cancer prevention benefit (55). Fourth, the impact of false-positive alerts on clinical workflow has been investigated in several recent studies, but consensus on mitigation strategies remains lacking.

Most studies have treated AI as a binary tool (on/off), but true human-machine collaboration involves more nuanced aspects: how clinicians interact with AI, how they develop trust in its recommendations, and under what circumstances they choose to override them. The eye-tracking dataset by Zhu et al. (60) showed that CADe universally shortened polyp detection reaction time, with a pronounced benefit for less experienced operators. A growing body of research has begun to investigate the impact of false-positive alerts on endoscopist behavior. Van Berkel et al. conducted a controlled lab study with 21 endoscopists and found that false-positive AI recommendations significantly prolonged decision-making time and that the type of medical content affected how quickly endoscopists responded to AI suggestions (61). Moreover, recent systematic reviews have identified false-positive signals as a major barrier to clinical adoption of AI systems, with endoscopists expressing concerns about workflow interruptions and alarm fatigue (62). In a prospective study on trust in CADx, inappropriate trust—including both overtrust and undertrust—was observed in 15.4% of diagnoses, with overtrust significantly lower among endoscopists with higher CRC expertise (74). Furthermore, emerging evidence raises concerns about potential skill degradation following prolonged AI exposure. Budzyń et al. found that the ADR of standard non-AI-assisted colonoscopy decreased significantly from 28.4% before AI implementation to 22.4% after (absolute difference −6.0%; *p* = 0.0089), suggesting that continuous exposure to AI might negatively affect endoscopist behavior when AI is not in use (75). This finding has been corroborated by others who argue that automation bias could erode diagnostic and technical performance over time unless mitigated through appropriate training and quality assurance (76). Additionally, no unified framework has yet been established for describing different models of human-AI collaboration in colonoscopy. Campion et al. proposed a framework categorizing key human factors including automation bias, alarm fatigue, algorithm aversion, learning effects, and deskilling (77). However, no empirical studies have yet compared different interaction frameworks (e.g., overlay alerts vs. side-by-side displays; CADe vs. CADx) in controlled trials, and consensus on optimal interaction design remains to be established.

The introduction of AI systems also alters the endoscopy workflow in tangible ways. Real-time alerts may distract from other procedural tasks, the physical placement of hardware requires additional space and setup, and documentation of false-positive findings can increase non-clinical workload. To date, no study has quantitatively assessed the impact of AI on overall procedural efficiency (e.g., total procedure time, interruption frequency) or on endoscopist cognitive load. Such evaluations are necessary to understand whether the gains in ADR come at the expense of workflow ergonomics and operator well-being. Beyond workflow considerations, ethical and regulatory issues surrounding AI in colonoscopy remain insufficiently addressed. AI systems are medical devices that require regulatory approval (e.g., FDA, CE marking), yet current approvals are based on short-term surrogate endpoints such as ADR rather than patient-relevant outcomes like CRC incidence or mortality (11). Moreover, data privacy, liability for missed lesions when AI fails, and algorithm drift over time are unresolved concerns. The “black-box” nature of deep learning models may hinder transparency and informed consent, making it difficult for patients and clinicians to fully understand the basis of AI-generated recommendations. These ethical and regulatory gaps must be closed before widespread deployment can be recommended.

A critical appraisal of the AI models reviewed reveals substantial heterogeneity in dataset size, validation strategies, and risk of overfitting. For example, the bowel preparation model by Zhou et al. (26) achieved 93.33% accuracy on only 120 images—a small dataset where overfitting cannot be ruled out. In contrast, the cloud-based CADe system in the EAGLE trial (59) was validated on 841 patients across eight centers, providing much stronger evidence for generalizability. Many single-center studies lack external validation, which limits their applicability to different populations, equipment, and bowel preparation protocols. Furthermore, most AI models reviewed were developed and validated using retrospective, high-quality image datasets, which may not fully represent real-world clinical conditions where bowel preparation quality varies and motion artifacts are common. Only a minority of studies [e.g., the EAGLE trial (59) and the GI Genius trial (57)] employed prospective, multicenter designs with real-time AI deployment. Additionally, the optimal performance threshold for CADe and CADx systems—balancing sensitivity against false-positive rates—has not been standardized, complicating cross-study comparisons. Some metrics reported in the literature, such as the silhouette coefficient [0.8116 and 0.6673 in Wang et al. (67)], are measures of clustering quality rather than direct clinical performance. While these indicate internal model consistency, they do not inform diagnostic accuracy or patient management; clinicians should focus on lesion-level sensitivity, specificity, and AUC, which were also reported and show strong performance. Moreover, the AI literature itself is susceptible to publication bias, with studies reporting positive results (e.g., high ADR improvements) being more likely to be published than those showing neutral or negative outcomes. This may inflate perceived effect sizes of AI systems in the literature. It is also important to acknowledge the limitations of the present review itself. As a narrative (non-systematic) review, it may suffer from selection bias (preferentially citing positive studies), a lack of quantitative synthesis, and subjective conclusions. Publication bias is likely, as negative or neutral results on AI-assisted colonoscopy are under-reported.

Looking forward, future research should prioritize several key areas: large, multicenter, real-world studies with long-term follow-up for interval CRC; standardized reporting of AI model training details (dataset size, validation methods, computational requirements); dedicated human-factors research on trust, reliance, and skill retention; rigorous cost-effectiveness analyses; and the development of integrated multifunctional AI platforms that cover the entire colonoscopy pathway—from bowel preparation assessment to post-polypectomy surveillance. Only through such comprehensive efforts can the promise of AI in colonoscopy be translated into tangible improvements in patient outcomes.

## Conclusion

In conclusion, the integration of artificial intelligence into colonoscopy has demonstrated promising and consistently observed benefits across multiple key domains, including bowel preparation assessment, cecal landmark identification, withdrawal quality monitoring, lesion detection, and polyp characterization. Large-scale randomized controlled trials and meta-analyses have consistently shown that AI-assisted colonoscopy significantly improves adenoma detection rate and reduces adenoma miss rate, particularly for diminutive and flat lesions, thereby addressing a major limitation of conventional endoscopy. Furthermore, AI enhances procedural standardization and reduces inter-observer variability, effectively narrowing the diagnostic gap between less experienced and expert endoscopists. These advances position AI as a powerful auxiliary tool that is already transforming clinical practice in digestive endoscopy.

Nevertheless, several important challenges remain to be addressed before AI can be universally adopted. Long-term outcome data linking AI-improved adenoma detection to a reduction in interval colorectal cancer incidence and mortality are still lacking. The dynamics of human-machine collaboration—particularly how endoscopists develop trust, handle false positives, and avoid skill degradation—require further investigation, and emerging evidence now indicates that skill degradation may indeed occur after prolonged AI exposure. Moreover, the clinical workflow integration, cost-effectiveness, and regulatory/ethical frameworks of AI systems need to be rigorously evaluated. Addressing these gaps through large-scale, multicenter, real-world studies and standardized reporting will be essential for translating the current technological promise into tangible, sustained improvements in patient outcomes. With continued refinement and validation, AI is poised to become an indispensable component of high-quality colonoscopy, ultimately contributing to more effective early screening and prevention of colorectal cancer.
